# Effect of preoperative hospital stay on surgical site infection in Chinese cranial neurosurgery

**DOI:** 10.1186/s12883-023-03431-z

**Published:** 2023-11-17

**Authors:** Lina Yang, Fengqiong Yi, Zhongyu Xiong, Huawen Yang, Yanchao Zeng

**Affiliations:** 1 Department of operating room nursing, Sichuan Provincial People’s Hospital, University of Electronic Science and Technology of China, Chengdu, 610072 China; 2 Department of Anesthesia and Surgery Center, The First Affiliated Hospital of Chongqing Medical University, 1st Youyi Road, Yuzhong District, Chongqing, 400016 China

**Keywords:** Craniotomy, Postoperative Complications, Risk factors, Surgical procedures

## Abstract

**Objective:**

Surgical site infection(SSI)after neurosurgical procedure can be devastating. Delayed hospital stay has been identified as a potentially modifiable driver of SSI in general surgery patients. However, the relationship between preoperative length of stay and SSI has not been quantified previously in neurosurgery. This study aimed to clarify the association.

**Design:**

A Cohort study based on STROBE checklist.

**Method:**

This observational study focused on cranial neurosurgery patients at a tertiary referral centers in China. Data collection from hospital information system conducted between 1 January 2016 and 31 December 2016 was used to examine the results of interest (n = 600). Logistic regression analysis explored association between preoperative length of stay and SSI, adjusting for potential confounders.

**Results:**

Overall SSI prevalence was 10.8% and was significantly higher in the longer preoperative length of stay group. Besides preoperative length of stay, American Society of Anesthesiologists score, type of surgery, gross blood loss also significantly associated with SSI prevalence. Compared with 1 to 2 days, longer preoperative length of stay was associated with increased SSI prevalence after adjustment for confounders (3 to 4 days: odds ratio[OR], 0.975[95%CI, 0.417 to 2.281]; 5 to 6 days: OR, 2.830[95%CI, 1.092 to 7.332]; 7 or more days: OR, 4.039[95%CI, 1.164 to 14.015]; P for trend < 0.001). On the other hand, we found a positive association between preoperative length of stay to deep/space-organ SSI (OR = 1.404; 95% CI: 1.148 to 1.717; P for trend < 0.001), which was higher than superficial SSI (OR = 1.242; 95% CI: 0.835 to1.848; P for trend= 0.062).

**Conclusions:**

In a cohort of patients from a single center retrospective surgical registry, a longer preoperative length of stay was associated with a higher incidence of cranial neurosurgical SSI. There is room for improvement in preoperative length of stay. This can be used for hospital management and to stratify patients with regard to SSI risk.

## Introduction

Length of stay (LOS) is defined as the number of days a patient resides in a facility from the day of admission till discharge [1]. In an effort to try to control health care costs, LOS is widely used as a key performance indicator in developed countries to assess hospital efficiency. In fact, LOS varies between countries. According to the Organization for Economic Cooperation and Development database (2019), the hospital average length of stay for diseases of nervous system in Canada is 14.2 days, while in France the LOS is 6.1 days. Prolonged preoperative length of stay seems to be the most common scenario in neurosurgery in low- and middle-income countries. A preoperative MRI examination is required, then surgery may be delayed. Inadequate blood preparation, waiting for the surgeon, and the operating room availability, can also lead to a surgical delay.

Previous studies link the length of stay with an increased cost of care, and reduced patient satisfaction [2, 3]. There has also been a growing interest among policymakers in the influence of prolonged preoperative hospital stays on several adverse postoperative outcomes, such as surgical site infection. Reported incidence of neurosurgical infection varies from 1%~8% in cranial cases [3–7]. Identifying preoperative risk factors for surgical site infection(SSI) helps clinical staff to respond. The factors that have been reported are as follows: age, sex, duration of operation, antibiotic prophylaxis, steroid use, American Society of Anesthesiologists (ASA) score, and so forth [8, 9]. Although there has been a great effort to improve the procedure quality, neurosurgical site infection is still possible. Therefore, it is quite necessary to evaluate the high-risk factors of SSIs. There are only a small number of studies examining the role of preoperative length of stay on SSIs in neurosurgical patients [10, 11].

In the special environment of hospitals, pathogens are more complex than those in conventional public places. There are many types of bacteria in the inpatient departments, especially Gram-negative drug-resistant bacteria. Prolonged preoperative length of stay increases the chance of bacterial colonization in patients. It was observed that the rate of Gram-negative colonization of oropharyngeal specimens increased during hospitalization in ICU and general ward patients, and the frequency of E. coli resistance increased significantly with the length of ICU stay [9]. But bacterial colonization is not the same as infection. The bacterial colonization theory has not convinced all scholars that preoperative hospital length of stay should be an independent risk factor for postoperative infection. After all, a longer preoperative hospital stay may also mean a more severe degree of illness and more co-morbid conditions for surgical patients.

In the literature review, studies on preoperative hospital stay and surgical site infection are mostly retrospective. Randomized controlled trials are difficult to implement, mainly for ethical reasons. One study showed that the variable length of preoperative hospital stay greater than 24 h was associated with approximately twice the chance of developing SSI, when compared to a length of hospital stay less than 24 h [12]. It is worth emphasizing that preoperative length of stay has been found in the literature as a risk factor for SSI in general surgery [13–15], but the same conclusion has not been reached in procedures such as orthopaedics [16]. Although preoperative length of stay has also been taken into account in some neurosurgery SSI studies, but most studies assessed specific subgroups of patients (e.g., elective surgery) or specific infections (e.g., meningitis) [10; 17, 18], and the specific number of days of LOS is less well understood.

We conducted a retrospective cohort study to examine the association of preoperative LOS on SSI after cranial neurosurgery. The strengths of this study are the size of the sample and the dose-response relationship of preoperative hospital stay to surgical site infection. This information should help the surgical team in optimizing their perioperative patient management.

## Methods

### Design

A retrospective review of adults (age ≥ 18 years) patients consecutive enrolled neurosurgery department between 1 January 2016 and 31 December 2016 was performed in a large general hospital. This 3200-bed tertiary hospital serves a total population of about 2,800,000 people with 1000 neurosurgical procedures performed per year. This cohort study was approved by the Institutional Review Committee of the hospital in which it was conducted.

### Patient selection

We included standard elective cranial neurosurgical procedures such as trepanation, craniotomy/craniectomy, cranioplasty, transsphenoidal surgery, and ventriculoperitoneal shunt insertion. Spinal procedures were not involved in this study. Patients with sepsis present at the time of surgery, superficial SSI, deep/organ space SSI, pneumonia, and urinary tract infection were excluded because of another potential underlying etiology. Patients with missing values (height, weight, age, preoperative length of stay, comorbidities, operation duration) and unknown values (ASA class, operation duration, gross blood loss) were also excluded.

### Patient characteristics and variables

The medical records, imaging findings, pathohistological reports, and microbiology results of the patients were extracted from the computerized hospital information system.

Patient demographics, comorbidities, ASA class, body mass index (BMI), preoperative length of hospital stay, wound classification, and operative data (laminar flow, operation duration, intraoperative blood transfusion, use of drain, implants et al.) were documented.

### Clinical routine and definition of SSI

Prior to the procedure, the patient’s head was shaved (neuro endoscopic transnasal pituitary adenoma resection is an exception). All patients receive a dose of antibiotics intravenously 30 to 60 min before surgery. The usual antibiotic is 1.5 g of cefuroxime. If the patient had a history of drug allergy, clindamycin 0.3 g was applied. Patients whose procedure lasted more than 3 h or who lost more than 1500 ml of blood intraoperatively were given another dose of antibiotics, with the aim of reducing the likelihood of wound infection from the neighboring bacterial skin flora.

The diagnostic criteria for SSI were based on the “Technical Guidelines for Surgical Site Infection Prevention and Control” and “Diagnosis of Nosocomial Infections” promulgated by the Chinese National Health Commission [19]. That was, when there was no prosthetic material, infections of the subcutaneous tissue, incisional skin, fascia, and muscle layer within 30 days after surgery were considered to be surgical site infections. When prosthetic material was present, infections occurring 1 year after surgery were also considered to be SSIs. Metal plates and screws used to fix the bone flap were considered prosthetic material. Ward nurses routinely followed patients up 30 days after the last craniotomy. If implants were present, they were followed up for 1 year.

### Statistic analysis

We categorized preoperative length of stay as 1 to 2 days, 3 to 4 days, 5 to 6 days, and 7 or more days. Conditional logistics regression analysis was used to estimate odds ratios (ORs) and 95% confidence intervals (CIs) for the association between the preoperative length of stay and the risk of SSI. In the logistic regression model, only the cohort of subjects with complete data in all variables was considered.

The logistic models were first adjusted for strata variables of age, gender at cohort entry. They were further adjusted for preoperative variables. Preoperative variables included BMI, ASA (II, III, IV), type of surgery (tumor 1, vascular 2, others 3), and Charlson comorbidity index(CCI) ( 0, 1, 2, 3) [20, 21]. The logistic models were then further adjusted for the perioperative covariables in the log-linear component: laminar flow (cleanliness class100 1, cleanliness class10,000 2), surgical duration(in hours), Infratentorial (0 or 1), wound class (clean 1, clean-contaminated 2), estimated blood loss (lg transformed), blood transfusion (0 or 1), implants (mental 1, mixed 2), and wound drain(0 or 1).

We did subgroup analysis by the outcome. The patient cohort was further divided into two groups: (1) patients with deep/organ-space SSI versus no SSI and (2) patients with superficial versus no SSI. Results are reported as ORs and CIs. For continuous variables, these refer to a per unit increase. Categorical variables, they refer to the comparison with a reference level, generally the most frequent category of the variable. Multicollinearity was assessed using the Pearson correlation coefficient statistic and by checking the Variance Inflation Factor on a multiple regression model with the same dependent and independent variables. All statistical tests of the hypothesis were two-sided and performed at the 0.05 level of significance. All analysis was performed using SPSS, version 22.0.

## Results

### Baseline characteristics of study participants, by preoperative length of stay

**Fig. 1:**
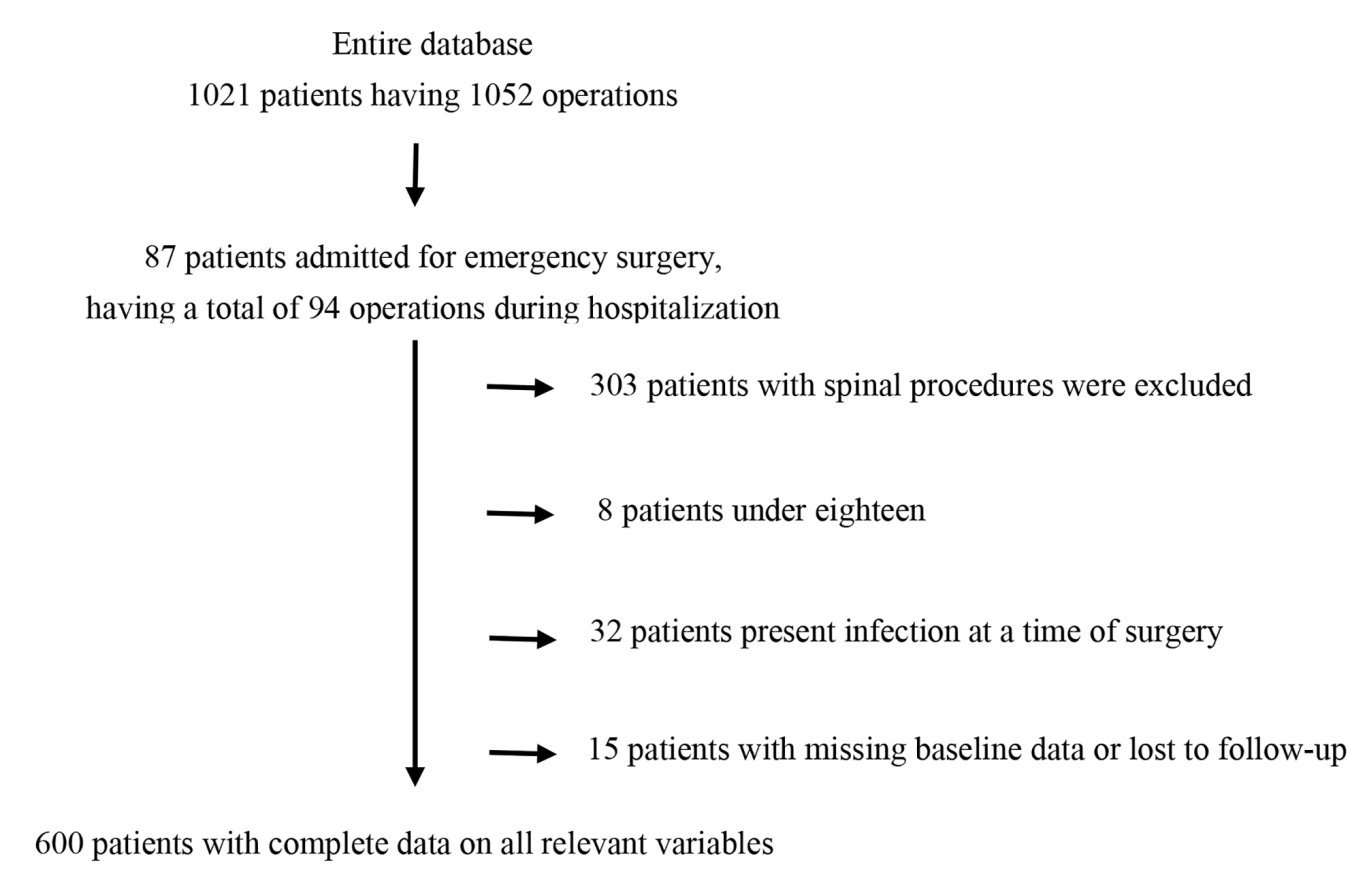
Summary of patient selection

1021 patients underwent at least one neurosurgical procedure at our hospital in 2016. Of these, four hundred and twenty-one were excluded from the study (Fig. 1). Consequently, a total of 600 procedures were selected for inclusion in the cohort. About 25% of patients were admitted to the hospital 1–2 days before surgery. More than half of patients stayed 3-4days preoperatively (Table 1). Patients with greater preoperative length of stay were more likely to be older, had longer surgical duration, and were more likely to receive intraoperative blood transfusions. There was a weak correlation between longer preoperative length of stay and higher CCI (r = 0.216). 76% of participants with the greatest preoperative length of stay group were identified as having Charlson score of 3 or more.

**Table 1:** Characteristics of participants in the cohort, by preoperative length of stay (n = 600)

Variable	Preoperative length of stay			
	1–2 days	3–4 days	5–6 days	≥ 7 days
Participants, n	144	352	76	28
Mean Age(SD),y	50.56(13.52)	50.64(13.35)	50.57(11.43)	50.75(14.35)
Female,%	49.3	54.5	60.5	75.0
Mean BMI(SD),kg/m^2^	22.78(3.05)	23.31(3.23)	23.41(3.26)	22.8(3.40)
CCI,%				
0	30.6	4.3	6.6	7.1
1	23.6	23.9	30.3	17.9
2	45.1	66.2	59.2	67.9
≥3	0.7	5.7	3.9	7.1
Type of surgery,%				
tumor	24.3	24.1	31.6	14.3
vascular	47.1	73.6	65.8	85.7
others	28.5	2.3	2.6	0.0
ASA,%				
II	17.4	1.1	2.6	7.1
III	39.6	39.5	32.9	42.9
IV	43.1	59.4	64.5	50.0
Laminar flow,%				
cleanliness class 100	42.4	52.0	46.1	46.4
cleanliness class 10,000	57.6	48.0	53.9	53.6
Mean Surgical duration (SD),h				
	3.39(1.86)	4.12(1.99)	4.39(2.33)	4.63(2.41)
Infratentorial,%	9.0	11.9	21.1	17.9
Wound class,%				
clean	85.4	76.4	81.6	82.1
clean-contaminated	14.6	23.6	18.4	17.9
Mean Ln Estimated blood loss (SD), ml				
	2.19(0.34)	2.35(0.39)	2.40(0.46)	2.42(0.48)
Blood transfusion,%	3.5	12.8	14.5	10.7
Implants,%				
mental	28.5	41.5	35.5	39.3
mixed	71.5	58.5	64.5	60.7
Wound drainage,%	54.2	73.9	76.3	75.0

Note: BMI: Body mass index; CCI: Charlson comorbidity index; ASA: American Society of Anesthesiologists

### Association of preoperative length of stay with SSI

Of these 600 procedures performed, 65 resulted in postoperative infection (in 61 patients), resulting in an infection rate of 10.8%. 4 patients developed both superficial and deep/organ-space surgical site infections. In the age-and sex-adjusted analysis, patients with ≥ 7 days of preoperative length of stay were associated with a higher risk for total SSI (Table 2). After adjustment for the surgical duration and other covariates, we saw an association between increasing preoperative length of stay and total SSI still existed. The OR for total SSI (vs. 1–2 days) was 0.975 (95%CI, 0.417 to 2.281) for 3–4 days, 2.830 (95%CI, 1.092 to 7.332) for 5–6 days, and 4.039 (95% CI, 1.164 to 14.015) for 7or more days. Positive associations were found in all subgroups, with a stronger association in deep/organ-space SSI participants (P for trend < 0.001).

**Table 2:** Preoperative length of stay and surgical site infection (SSI) in the cohort

Preoperative length of stay (day, media[range])	Partici-pants, n	SSI, n	Adjusted Odd Ratio(95%CI)		
			Model 1 *	Model 2†	Model3‡
**Total**					
Q1(2 [1, 2])	144	9	1.000(reference)	1.000(reference)	1.000(reference)
Q2(3 [3, 4])	352	27	1.254(0.574–2.739)	0.913(0.408–2.041)	0.975(0.417,2.281)
Q3(5 [5, 6])	76	18	4.714(1.994–11.142)	3.548(1.456–8.644)	2.830(1.092,7.332)
Q4(7[≥ 7])	28	7	5.141(1.711–15.445)	4.721(1.496–14.899)	4.039(1.164,14.015)
P for trend			0.000	0.000	0.001
Increase per day			1.423(1.211–1.672)	1.428(1.196–1.706)	1.387(1.147,1.677)
**Deep**§					
Q1(2 [1, 2])	144	6	1.000(reference)	1.000(reference)	1.000(reference)
Q2(3 [3, 4])	352	26	1.856(0.747–4.613)	1.354(0.533,3.442)	1.590 (0.598,4.223)
Q3(5 [5, 6])	76	13	4.864(1.762–13.428)	3.544(1.248,10.069)	2.867(0.948,8.668)
Q4(7[≥ 7])	28	7	8.106(2.452–26.796)	7.652(2.193,26.707)	7.522(1.909,29.639)
P for trend			0.000	0.000	0.001
Increase per day			1.462(1.233–1.733)	1.461(1.209–1.766)	1.404(1.148,1.717)
**Superficial**||					
Q1(2 [1, 2])	144	3	1.000(reference)	1.000(reference)	1.000(reference)
Q2(3 [3, 4])	352	3	0.408(0.081,2.054)	0.267(0.050,1.424)	0.281(0.045,1.770)
Q3(5 [5, 6])	76	6	4.173(1.002,17.373)	2.818(0.646,12.296)	2.474(0.481,12.725)
Q4(7[≥ 7])	28	1	1.797(0.176,18.368)	1.332(0.127,13.929)	1.394(0.111,17.513)
P for trend			0.020	0.030	0.062
Increase per day			1.276(0.922–1.765)	1.296(0.903–1.861)	1.242(0.835,1.848)

*Adjusted for age and gender at cohort entry

† The following variables were also included to control for the effect of body mass index, Charlson comorbidity index, type of surgery and American Society of Anesthesiologists score

‡ Further adjusted for laminar flow, surgical duration, infratentorial, wound class, estimated blood loss, blood transfusion, implant and wound drain

§ Excludes deep/organ-space infection

|| Excludes superficial infection

## Discussion

SSIs have a negative impact on patients with cranial neurosurgery. The incidence of SSI in our study was as high as 10.8%. SSIs not only prolong hospitalization days but also increase mortality rates. Given these consequences, reduction in the incidence of SSI has become a central theme of quality improvement in healthcare institutions. Guideline-oriented surgical site infection improvement program has been proven effective in some vascular surgery [22]. However, their efficacy in cranial neurosurgery is poorly defined. Dedicated strategies to improve neurosurgical site infection are limited.

In our analysis, patients were admitted 1–10 days before elective cranial neurosurgery was carried out. The logistic regression model showed that preoperative length of stay and SSIs are associated. In addition, the preoperative ASA score was related to SSIs [23–25]. Others identified perioperative variables, such as type of surgery, and gross blood loss [26, 27], that were relevant to SSIs. Those were also included and confirmed in our analysis.

Association between preoperative length of stay and SSI has been reported in previous studies. A retrospective cohort study noted that a length of preoperative hospital stay longer than four days was identified for SSI with femoral fracture patients [28]. Despite this, guidelines published by Centers for Disease Control for prevention of surgical site infection suggest that preoperative inpatient hospitalization is a surrogate for other risk factors. While our study confirmed the association between preoperative length of stay and SSIs among patients underwent cranial neurosurgery. The association remained significant after adjusting for inpatient status and operative factors. It is worthwhile to note that preoperative length of stay is a relatively interventional factor. For example, failure to undergo examination or inadequate preoperative conversation may lead to a delayed hospital stay. However, the identification of an association between preoperative length of stay and SSIs does not necessarily imply that shorter preoperative hospitalization leads to a decreased SSI rate. Prospective studies will be required to develop evidence to guide patient management strategies.

Our findings of increased risk of SSI with prolonged preoperative length of stay are consistent with cohort studies conducted in Japan [13], Brazil [12], and Ethiopia [29]. In our study, the risk of SSI increased by 183.0% for preoperative hospitalization with 5–6 days and by 303.9% for preoperative hospitalization with more than 7 days. This is similar to previous reports from large Japanese cohorts. An increased SSI risk of 112% was seen in general surgical procedures with 1–2 days of preoperative length of stay, compared to same-day surgery. The Ethiopian study reported that a preoperative hospital stay of more than 7 days added the risk of SSI by 22.44 times compared with less than 7 days. Nonetheless, no association was found between preoperative length of stay and SSIs in US data [16]. Such investigations are important because patterns of care and disease risks may vary across areas. Findings in one group may not necessarily apply to others. In our study, 34.8% of patients were admitted to the hospital 3 days before surgery. No sample underwent same-day surgery. We observed the increased risk in the group with ≥ 7 days preoperative length of stay did not as significant as in other studies, likely due to fewer participants in that group.

Hospitals are places gathering various pathogenic bacteria. The longer the hospital stay, the more exposure to pathogenic bacteria. A study exploring the effect of surgical delay on bacterial colonization [30] confirmed that, both the number of colony-forming units and individual species p.a. pathogen bacteria increased from day 0 to day 5. When we modeled separately to explore the role of preoperative length of stay in superficial and deep/organ-space infection, an interesting finding emerged. Delayed preoperative length of stay increased the odds of all surgical site infection types. The graded relationship between the preoperative length of stay and deep/organ-space infection seems to be stronger. Patients with ≥ 7 days preoperative hospitalization suffered 652.2% higher risk for deep/organ-space infection and 39.4% higher risk for superficial infection than with 1–2 days preoperative hospitalization. This may be related to the application of implants in cranial neurosurgery. Quantitative studies have shown that the dose of contaminating microorganisms required to produce infection may be much lower when foreign material is present at the site [31; 32]. In our study, implants were placed in all 600 procedures, either metallic or mixed. These implants provided a nucleus for organism attachment. Even low doses of contaminating microorganisms have the potential to induce deep/organ-space infection. Preoperative length of stay may interact with other factors to form new pathways that amplify the risk of SSI. It requires further investigation.

The strengths of this study include suitable sample size, standardization of the procedures used to collect risk factor information, and limited loss to follow-up. This is also the only study to date that has explored whether the association between preoperative LOS and SSIs differs by subtypes in cranial neurosurgery.

## Conclusion

After adjustment for preoperative co-morbidity and surgical complexity as risk factors, preoperative length of stay remains important in predicting SSI. Thus, preoperative length of stay is a potentially modifiable variable for improving clinical outcomes of patients with cranial neurosurgery.

### Limitations

The present study has limitations. First, this was a single-center study and statistical analyses were retrospective. The results may not be generalizable to other institutions. Second, the associations we observed between SSI and risk factors do not necessarily indicate causality, although other studies have reported similar correlations, as outcomes may still be influenced by unmeasured confounders. Third, we did not record all risk factors documented in other studies, such as tobacco use ever, steroid treatment, and preoperative transfusion. Despite these limitations, our findings may provide useful reference data for the management of infection control and treatment.

## Data Availability

The original contributions presented in the study are included in the article material, further inquiries can be directed to the corresponding authors.
